# Clonogenicity-based radioresistance determines the expression of immune suppressive immune checkpoint molecules after hypofractionated irradiation of MDA-MB-231 triple-negative breast cancer cells

**DOI:** 10.3389/fonc.2023.981239

**Published:** 2023-04-20

**Authors:** Simon Gehre, Felix Meyer, Azzaya Sengedorj, Fridolin Grottker, Clara M. Reichardt, Jannik Alomo, Kerstin Borgmann, Benjamin Frey, Rainer Fietkau, Michael Rückert, Udo S. Gaipl

**Affiliations:** ^1^ Translational Radiobiology, Department of Radiation Oncology, Universitätsklinikum Erlangen, Friedrich-Alexander-Universität Erlangen-Nürnberg, Erlangen, Germany; ^2^ Department of Radiation Oncology, Universitätsklinikum Erlangen, Friedrich-Alexander-Universität Erlangen-Nürnberg, Erlangen, Germany; ^3^ Comprehensive Cancer Center Erlangen-EMN, Erlangen, Germany; ^4^ Laboratory of Radiobiology and Experimental Radiooncology, Department of Radiotherapy and Radiation Oncology, Center of Oncology, University Medical Center Hamburg-Eppendorf, Hamburg, Germany

**Keywords:** radiotherapy, breast cancer, radioresistance, immune checkpoint molecules, dendritic cells, tumor cell death

## Abstract

Only a subset of patients with triple-negative breast cancer (TNBC) benefits from a combination of radio- (RT) and immunotherapy. Therefore, we aimed to examine the impact of radioresistance and brain metastasizing potential on the immunological phenotype of TNBC cells following hypofractionated RT by analyzing cell death, immune checkpoint molecule (ICM) expression and activation of human monocyte-derived dendritic cells (DCs). MDA-MB-231 triple-negative breast cancer tumor cells were used as model system. Apoptosis was the dominant cell death form of brain metastasizing tumor cells, while Hsp70 release was generally significantly increased following RT and went along with necrosis induction. The ICMs PD-L1, PD-L2, HVEM, ICOS-L, CD137-L and OX40-L were found on the tumor cell surfaces and were significantly upregulated by RT with 5 x 5.2 Gy. Strikingly, the expression of immune suppressive ICMs was significantly higher on radioresistant clones compared to their respective non-radioresistant ones. Although hypofractionated RT led to significant cell death induction and release of Hsp70 in all tumor cell lines, human monocyte-derived DCs were not activated after co-incubation with RT-treated tumor cells. We conclude that radioresistance is a potent driver of immune suppressive ICM expression on the surface of TNBC MDA-MB-231 cells. This mechanism is generally known to predominantly influence the effector phase, rather than the priming phase, of anti-tumor immune responses.

## 1 Introduction

Triple-negative breast cancer (TNBC) is defined by the absence of estrogen receptor (ER), progesterone receptor (PR) and human epidermal growth factor receptor 2 (HER2) expression. It accounts for 10-20% of all breast cancer cases and is characterized by high invasiveness, early metastasis (esp. lung- and brain-metastases), and high recurrence rate. Despite similar therapeutic approaches (surgery, chemo- and radiotherapy), TNBC remains the breast cancer subtype with the worst prognosis. High heterogeneity, the lack of hormone receptors and chemoresistance (triple-negative paradox) leave little room for targeted therapy approaches (1–3). Therefore, therapeutic strategies leading to improved therapy outcomes are urgently needed.

Adjuvant radiotherapy (RT) is perceived as standard of care in patients with early-stage breast cancer undergoing breast-conserving surgery and complete mastectomy. The goal of it is to reduce the risk of locoregional recurrence and breast cancer associated mortality (4, 5). In this context moderately hypofractionated RT (HFRT) has gained importance over the last years (6). It is characterized by increased dose per fraction and simultaneously, decreased fractions in total (40 Gy in total, 15-16 fractions in 3-5 weeks). In comparison to conventional fractionation schedules (50 Gy in total, 25-28 fractions in 5-6 weeks), this approach offers lower acute toxic effects while maintaining local tumor control (7–9). Recently, the FAST-Forward trial indicated that a super-hypofractionated five-day treatment schedule of postoperative radiotherapy (26 Gy, five fractions in one week) is non-inferior to moderately hypofractionated adjuvant radiation therapy (40 Gy, 15 fractions in three weeks) in terms of local tumor control and side effects in women with early-stage breast cancer (10).

RT in general is attributed to both immune stimulatory and immune inhibitory effects. On the one hand, it can enhance anti-tumor immunity by cell death-triggered release of neoantigens, damage-associated molecular patterns (DAMPs, e.g. HMGB1, ATP, Hsp70) and proinflammatory substances (e.g. CXCL10 and CXCL16). Additionally, the activation of the cGAS/STING pathway including consequent type I interferon production and increased MHC-I expression for antigen presentation on the cell surface of cancer cells is also activated by RT (11). Besides the control of the immune checkpoint PD-L1/PD1 axis by interferons, also less well understood immune checkpoint molecules are triggered (12). Hypofractionated RT induces DNA damage and impaired DNA repair results in transfer and accumulation of DNA fragments in the cytoplasm of the tumor cells. As physiological mechanisms for detection of cytosolic DNA (e.g. resulting from invading pathogens), DNA sensing pathways as the cGAS/STING pathway are triggered that activate the innate immune response through a signaling cascade leading to upregulation of cytokine and interferon production (13). This is also a common mechanism in triple negative breast cancer that impacts on tumor cell survival and immune modulation (14, 15). On the other hand it was shown by Rückert et al., that HFRT in particular is predestined to induce immunogenic cell death (ICD) (16), which is defined as “a form of regulated cell death (RCD) that is sufficient to activate an adaptive immune response in immunocompetent syngeneic hosts” (17) leading to T cell-mediated immune responses against tumor antigens. Based on that, RT has been reported to work as *in situ* cancer vaccine making abscopal effects possible (18). On the other hand, RT can also mediate immune suppressive effects, for example by inducing an increased expression of immune suppressive immune checkpoint molecules (ICMs), the release of immune inhibitory cytokines (e.g. TGF-β) and the infiltration of T regulatory cells (Tregs) as well as myeloid derived suppressor cells (MDSC) into the tumor area (11). The T cell suppression in the effector phase of the immune response mediated *via* immune inhibitory ICM interactions, can be antagonized by immune checkpoint inhibitors (ICIs). Consequently, a tumor-antigen specific cytotoxic T cell response can be restored (19, 20). That makes combinations of radiotherapy and immune checkpoint blockade (ICB) reasonable.

Although breast cancer has been perceived historically as immunologically “cold” tumor, it becomes more and more evident that the different subtypes differ a lot regarding their respective immunogenicity. TNBC seems to be the most immunogenic subtype, because of its higher tumor infiltrating lymphocyte (TIL) counts and tumor mutational burden (TMB) (21). Supporting this, ICI therapy particularly benefits those breast cancer patients suffering from TNBC (22). Therefore, a growing number of clinical trials examining the efficacy of ICB in patients with TNBC have recently been conducted. Unfortunately, only a small minority of these patients has been shown to benefit from anti-PD-(L)1 monotherapy in terms of overall response rate (ORR) (23). However, Ho et al. reported that therapeutic approaches combining RT and ICB could be superior to ICI monotherapy (24). In this context radioresistant cancer cells remain a major challenge in TNBC treatment because of their capacity to form local- and distant recurrence.

In the past, radioresistance of a cell has always been defined based on the ability to form new cell colonies after being irradiated (25, 26). Since presumably radiation-resistant (breast) cancer cells are responsible for recurrence or metastasis after RT, it may not only be radioresistance alone but rather the combination with immune evasion allowing breast cancer cells to survive and form clinically apparent tumors. Therefore, we hypothesized that radioresistance itself could significantly drive the immunogenic properties of breast cancer cells. To investigate this for the first time, we treated two different radioresistant (RR) and two non-RR MDA-MB-231 cell lines with hypofractionated RT (5 x 5.2 Gy) and analyzed cell death induction by AnnexinV/Propidium iodide staining, Hsp70 release and the activation of human monocyte-derived dendritic cells (DCs) after previous co-cultivation. Furthermore, the immune checkpoint molecule expression on the tumor cell surface was examined. Our key finding was that the expression of immune suppressive ICMs was significantly increased in the radioresistant cell lines after RT.

## 2 Materials and methods

### 2.1 Cell lines and cell culture

**Figure 1:**
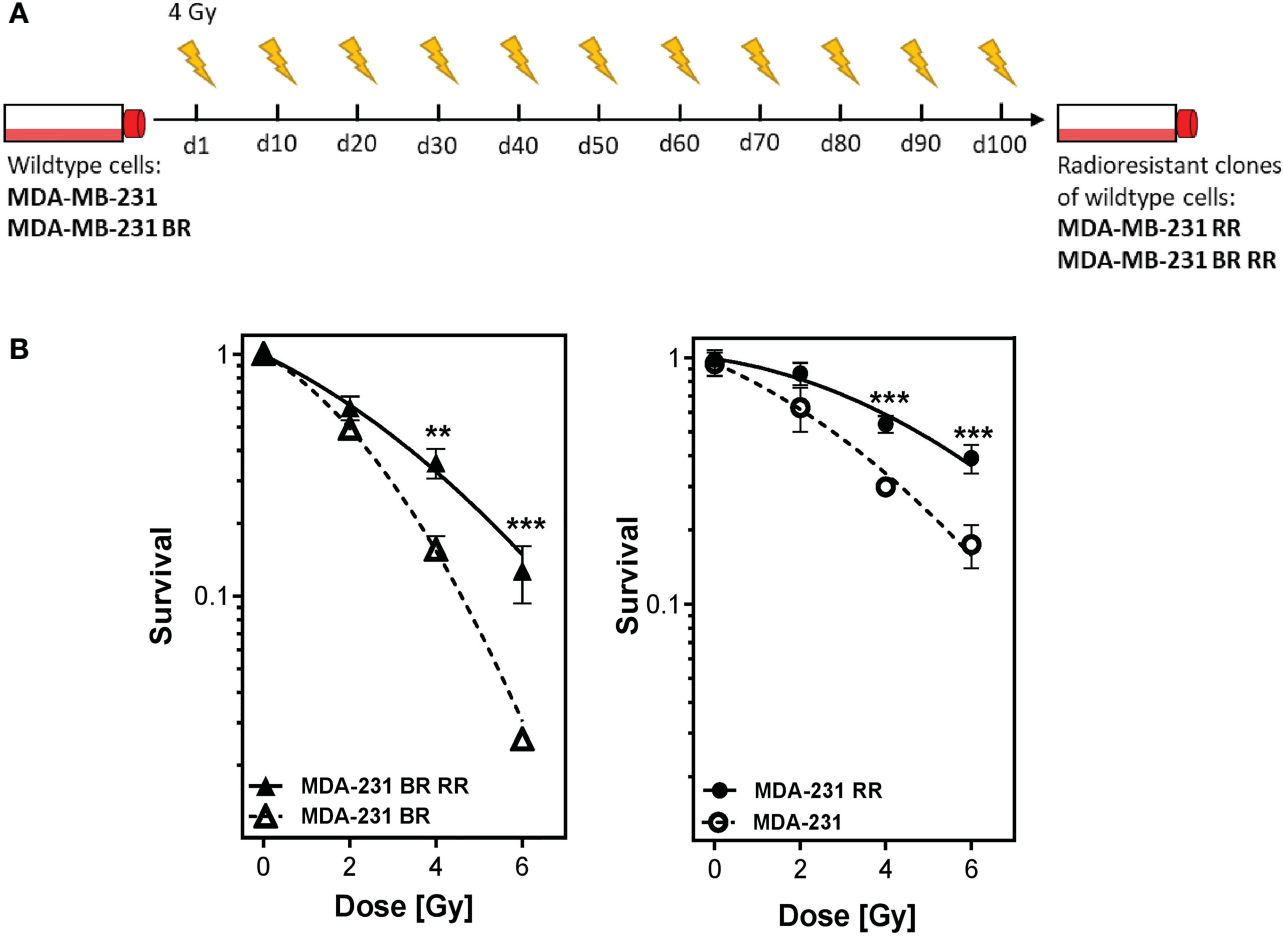
Generation of radioresistant breast cancer clones is done by repeated irradiation of MDA-MB-231 breast cancer cells. Radioresistant (sub)clones of MDA-MB-231 cells were generated by repeatedly irradiating MDA-MB-231 wildtype (MDA-MB-231) and brain metastasizing MDA-MB-231 (MDA-MB-231 BR) tumor cells **(A)**. This resulted in more radioresistant clones (MDA-MB-231 RR and MDA-MB-231 BR RR), as verified by clonogenic survival assay **(B)**. Data are from three independent experiments. **p < 0.01; ***p < 0.001 (Student’s t-test).

Four different human MDA-MB-231 cell lines with differences in radioresistance (according to their behaviour in the clonogenic assays) were investigated (27). Besides the wildtype (WT), a brain-metastasizing (BR) clone was used. It was created by Yoneda et al., 2001 by inoculating the MDA-MB-231 WT cells into immunodeficient mice. MDA-MB-231 cells in brain metastases were isolated, grown in culture and reinoculated. This procedure was repeated until only brain metastases occurred after injection into immunodeficient mice (28). Radioresistant (sub)clones (WT RR, BR RR) were generated by irradiation of the WT and BR clone with 4 Gy, pooling of the surviving cells, cultivating them for 10-14 days and irradiating them again. This procedure was repeated to a total dose of at least 40 Gy. Radioresistance was checked after the last irradiation with clonogenic assay (**Figures 1A, B**).

All four cell lines were cultivated in Dulbecco’s modified Eagle’s medium (DMEM, Pan-Biotech GmbH, Aidenbach, Germany) supplemented with 10% fetal bovine serum (FBS, Biochrom AG, Berlin, Germany) and 1% Penicillin-Streptomycin (PenStrep, Gibco, Carlsbad, CA, USA). Peripheral blood mononuclear cells (PBMCs) derived from healthy human donors were cultured in “DC medium” consisting of RPMI-1640 (Merck, Darmstadt, Germany) supplemented with 1% Pen/Strep, 1% L-Glutamine (Gibco, Carlsbad, CA, USA), 1% Hepes buffer (Gibco Life Technologies, Waltham, MA, USA) and 1% human serum heat inactivated (Gibco, Carlsbad, CA, USA). All cells were cultivated in a standardized and humidified environment (37°C, 5% CO_2_ and 95% humidity).

### 2.2 Treatments and sampling

The day after seeding, the four MDA-MB-231 cell lines were irradiated for five days with 5.2 Gy of X-rays (120 kV, 22.4 mA for 0.7 min; X-Ray tube Isovolt Titan, GE Sensing & Inspection, Boston, USA), respectively. The cells were harvested with trypsin (Gibco Life Technologies, Carlsbad, CA, USA) on day 6, 7 and 8 for cell death analysis, on day 7 for ICM expression analysis and on day 6 to evaluate the DC activation potential of untreated and treated tumor cells after co-incubation. Hsp70 concentration in the cell culture supernatant was determined 48 hours after irradiation (day 7) *via* ELISA.

### 2.3 Cell death analysis and clonogenic survival assay

2 × 10^5^ cells were stained with 100 μl of cell death staining solution (1 ml of Ringer’s solution (Fresenius Kabi, Bad Homburg, Germany), 0.75 μl/ml of AnnexinV-FITC (AxV) (1 mg/ml) (GeneArt, Regensburg, Germany), and 1.0 μl/ml of Propidium iodide (Pi) (1 mg/ml) (Sigma-Aldrich, Munich, Germany)). After incubation for 30 minutes, the measurement was performed on a CytoFLEX S flow cytometer (Beckman Coulter, Brea, CA, USA) and analyzed with the Kaluza Analysis software (Beckman Coulter, Brea, CA, USA).

To determine the clonogenic potential of the breast cancer cells, 250 tumor cells/well were seeded in a 6-well plate 6h before irradiation. Afterwards they were irradiated with the indicated doses and cultured for two weeks, fixed and stained with 1% crystal violet in ethanol (Sigma-Aldrich, St. Louis, MO). Colonies with more than 50 cells were counted and normalized to mock-treated samples. The survival curves were calculated by adding a curve fit (dek(hx)). All calculations were performed with GraphPad Prism.

### 2.4 Immune checkpoint molecule expression analysis

2 × 10^5^ cells were stained with staining solution containing FACS buffer (PBS (Sigma-Aldrich, Munich, Germany), 2% FBS and 4% 0.5 mM EDTA (Carl Roth, Karlsruhe, Germany)) and Zombie NIR alone or Zombie NIR and antibodies (**Table 1**). Before the measurement at the CytoFLEX S flow cytometer, the cells were incubated for 30 minutes at 4°C. To correct for treatment-related autofluorescence, the ΔMFI (mean fluorescence intensity) of every ICM was calculated by subtracting the MFI of the Zombie-only-stained sample (AF ctrl) from the MFI of the Zombie-and-antibody stained one.

**Table 1:** List of antibodies used to analyze the expression of immune checkpoint molecules on the surface of non-irradiated and irradiated MDA-MB-231 tumor cells *via* multicolor flow cytometry.

Marker	Fluorochrome	Manufacturer
PD-L1 (CD 274)	BV 605	Biolegend
PD-L2 (CD 273)	APC	Biolegend
ICOS-L (CD 275)	BV 421	BD Horizon
HVEM (CD 270)	APC	Biolegend
TNFRSF9 (CD137-L)	BV 421	Biolegend
OX40-L (CD252)	PE	Biolegend
Live/dead	Zombie NIR	Biolegend

### 2.5 Quantitative measurement of Hsp70 in the supernatant of untreated and treated MDA-MB-231 cells

To examine the concentration of tumor cell released Hsp70, the supernatant of the cell cultures was analyzed using a sandwich ELISA assay (Human/Mouse/Rat Total HSP70/HSPA1A ELISA, R&D Systems, Minneapolis, MN, USA). It was performed according to the manufacturer’s instructions.

### 2.6 Isolation of human peripheral blood mononuclear cells and differentiation to human monocyte-derived dendritic cells

On day 0, human peripheral blood mononuclear cells (PBMCs) were isolated from leukoreduction system chambers (LRSC) of healthy human donors *via* density gradient centrifugation in SepMate™ PBMC Isolation Tubes (Stemcell, Vancouver, Canada) and Lymphoflot (Biotest AG, Dreieich, Germany). Consequently, they were washed twice at 4°C with PBS (Sigma-Aldrich, Munich, Germany)/0.5 mM EDTA (Carl Roth, Karlsruhe, Germany) and RMPI-1640, respectively. After that, 30 × 10^6^ cells each were seeded on IgG-pre-coated cell culture dishes in 10 ml of DC medium and incubated for 1 h. Subsequently, non-attached cells were removed and 10 ml of fresh DC medium was added.

On day 1, the old DC medium was removed again and 10 ml of RPMI containing 800 U/ml (0.57 μl/ml) of GM-CSF (MACS Miltenyi Biotec, Bergisch Gladbach, Germany) and 500 U/ml (5 μl/ml) of IL-4 (ImmunoTools, Friesoythe, Germany) were added to each cell culture dish. Two days later, on day 3, 4 ml of RPMI and 800 U/ml (0.57 μl/ml) of GM-CSF and 500 U/ml (5 μl/ml) of IL-4 were added. On day 5, 4 ml of RPMI with half of the previously used amount of GM-CSF (400 U/ml = 0.285 μl/ml) and IL-4 (250 U/ml = 2.5 μl/ml) were added.

### 2.7 Maturation induction of immature DCs *via* maturation cocktail or co-incubation with untreated and treated MDA-MB-231 cell lines

Six days after the isolation of the PBMCs, the human monocyte-derived immature DCs (iDCs) were harvested mechanically using a serological pipette. After that, 0.75 × 10^5^ iDCs were put into each 6-well in 2 ml of DC medium. In case of co-incubation with non-irradiated or irradiated tumor cells, 1.5 × 10^5^ tumor cells including 2 ml of their respective cell culture supernatant were added. Positive controls (without tumor cells) were established by using a maturation cocktail (MC) containing 13.16 ng/ml of IL-1β (ImmunoTools, Friesoythe, Germany), 1000 U/ml of IL-6 (ImmunoTools, Friesoythe, Germany), 10 ng/ml of TNF-α (ImmunoTools, Friesoythe, Germany) and 1 μg/ml of PGE-2 (Pfizer, Berlin, Germany).

### 2.8 Maturation

The expression of common activation markers on the cell surface of the DCs was analyzed 48 hours after co-incubation with untreated and treated MDA-MB-231 cells using multicolor flow cytometry. Therefore, the DCs were harvested mechanically at first. Then, the first half of all DCs in each 6-well was stained with a Zombie-only FACS buffer staining solution, the second half was stained with one containing Zombie Yellow and antibodies in addition (**Table 2**). After incubation for 30 minutes at 4°C, the MFI of the different samples was measured at the CytoFLEX S flow cytometer. The ΔMFI of every activation marker was calculated by subtracting the MFIs of the Zombie-only from the fully stained sample.

**Table 2:** List of antibodies used to analyze the expression of various activation markers on the surface of DCs *via* multicolor flow cytometry.

Marker	Fluorochrome	Manufacturer
CD70	FITC	Biolegend
CD83	PE-Cy7	eBioscience
CD80	APC	Miltenyi Biotec (MACS)
CD86	Briliant Violet	BioLegend
Live/dead	Zombie Yellow	Biolegend

### 2.9 Statistical analyses

For statistical analyses the Student’s t-test, the Mann-Whitney U test and the Kruskal-Wallis test with multiple comparisons were used as respectively indicated in the figure legends.

## 3 Results

### 3.1 Radioresistant clones of MDA-MB-231 cells can be generated by repeated irradiation

MDA-MB-231 wild type and brain metastasizing tumor cells were repeatedly irradiated with 4 Gy to generate more radioresistant clones, as being verified by standard clonogenic survival assay (**Figure 1**). The four different human MDA-MB-231 cell lines, MDA-MB-231 WT, MDA-MB-231 BR (brain metastasizing) and the corresponding more radioresistant clones (RR) were used for the consecutive analyses.

### 3.2 Radiation-induced apoptosis of MDA-MB-231 cells depends on tissue origin of the tumor cells rather than on radioresistance

**Figure 2:**
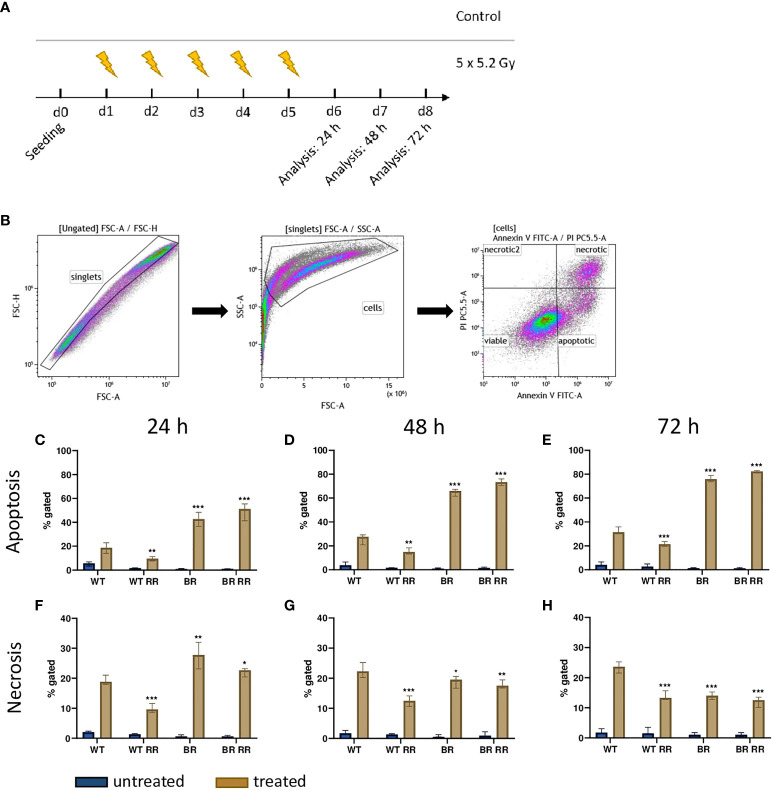
Cell death induction after irradiation of the four MDA-MB-231 cell lines is dependent on tissue origin rather than on radioresistance. **(A)** After seeding on day 0, the four MDA-MB-231 cell lines, the WT and the WT-derived brain metastasis clone (BR) as well as the radioresistant (RR) clones derived from those cells (WT RR, BR RR), were treated with 5 × 5.2 Gy. On day 6, 7 and 8, cell death forms were analyzed with Annexin V/Propidium iodide (AxVPi) staining *via* multicolor flow cytometry. The gating strategy is shown in **(B)**. After pre-gating on the singlets and consequently excluding the debris, the remaining cells were identified as viable, apoptotic, or necrotic as presented. The percentage of apoptosis **(C–E)** and necrosis **(F–H)** of the different cell lines 24 **(C–F)**, 48 **(D, G)** and 72 hours **(E–H)** after irradiation is shown as median with interquartile range. The data are from nine independent experiments. For statistical analysis, each treated clone was compared to the WT *via* Mann-Whitney U test (*p < 0.05, **p < 0.01, ***p < 0.001).

We then analyzed cell death 24 h – 72 h after hypofractionated irradiation of the four MDA-MB-231 tumor cell lines (**Figures 2A, B**). Apoptosis, rather than necrosis (**Figures 2C–E**), was the predominant cell death mechanism and, differed significantly between the irradiated WT and its clones. Apoptosis increased over time, whereas the ratio between the respective cell lines remained similar. Both cell lines that had been derived from brain metastases (BR, BR RR) were very radiosensitive in terms of apoptosis induction and succumbed to it significantly more frequent in comparison to the WT. BR RR even showed the greatest apoptosis rate of all four cell lines despite its radioresistance in the clonogenic assay (**Figure 1B**), closely followed by BR. This was different in the WT RR clone that was significantly less sensitive to irradiation with regard to apoptosis.

Similarly, necrotic cell death (**Figures 2F–H**) was significantly increased in cell lines derived from brain metastases (BR, BR RR) compared to the WT cell line 24 h after the treatment. Further, only the WT RR cell line showed significantly less necrosis than the WT. However, 48 and 72 hours after irradiation necrotic cell death was significantly decreased in all clones compared to the WT.

### 3.3 Radioresistance drives the expression of immune suppressive checkpoint molecules following irradiation

The expression of the investigated ICMs did not vary considerably between the untreated WT and its clones. There was a similar base level of ICM expression between all four different cell lines, with just one exception: the immune stimulatory ICM CD137-L was significantly lower expressed in all non-irradiated clones that were originally derived from the WT.

**Figure 3:**
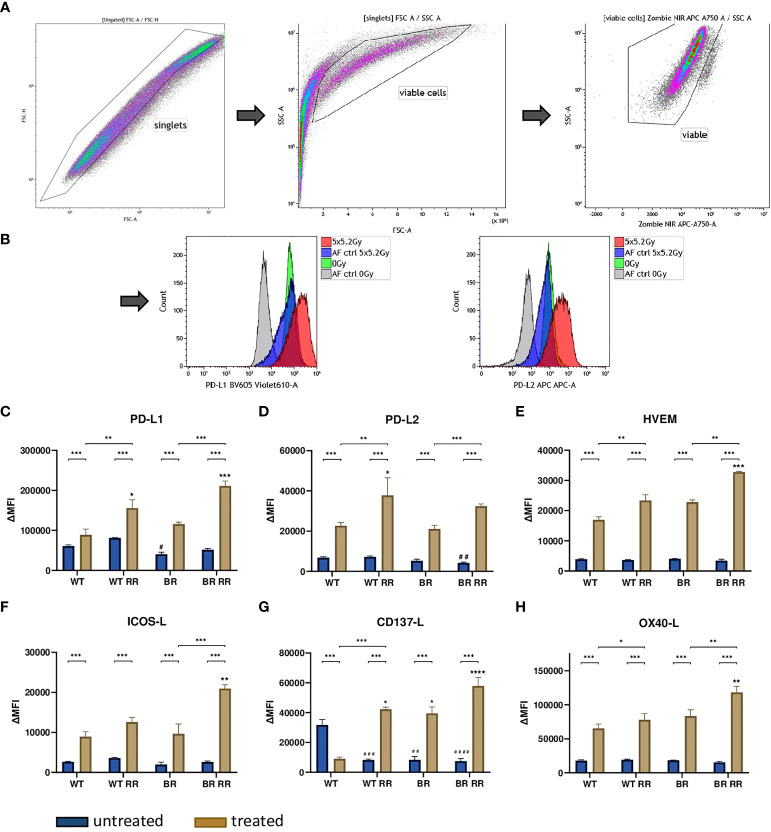
Radioresistance (RR) drives the expression of immune suppressive checkpoint molecules on the surface of the four presented MDA-MB-231 cell lines 48 hours after hypofractionated irradiation. The gating strategy is presented in **(A)** After pre-gating on the singlets, the debris was excluded. Then the viable cells were detected *via* the Zombie NIR viable/dead stain. Immune checkpoint molecule (ICM) expression is presented in the graphs as ΔMFI (mean fluorescence intensity). It was calculated by subtracting the MFI of the Zombie-only-stained samples (AF ctrl) from the respective Zombie-and-antibody-stained samples of various ICMs expressed on the cell surface of the four cell lines. Exemplarily primary data are shown for PD-L1 and PD-L2 detection. The WT and the WT-derived brain metastasis clone (BR) as well as the radioresistant (RR) clones derived from those cells (WT RR, BR RR) were treated with 5 × 5.2 Gy. **(B)** The expression of immune suppressive (PD-L1: **(C)**, PD-L2: **(D)**, HVEM: **(E)** and immune stimulatory (ICOS-L: **(F)**, CD137-L: **(G)**, OX40-L: **(H)** ICMs is presented as median with interquartile range. Data are from seven independent experiments. For statistical analysis, a Mann-Whitney U test was performed to compare untreated and treated cells within one cell line. The same test was used to compare an irradiated radioresistant cell clone with its respective non-radioresistant one. A Kruskal-Wallis test with multiple comparisons was used to examine statistical differences between the ICM expression of the different clones compared to the WT within the respective untreated (#) and treated (*) group. *p < 0.05, **p < 0.01, ***p < 0.001, ****p < 0.0001, ^#^p < 0.05, ^##^p < 0.01, ^###^p < 0.001, ^####^p < 0.0001.

Irradiation with 5 × 5.2 Gy resulted in a significant upregulation of both immune suppressive (PD-L1, PD-L2 and HVEM) (**Figures 3C–E**) and immune stimulatory (ICOS-L, CD137-L, OX40-L) (**Figures 3F–H**) immune checkpoint molecules on the cell surface of the treated cells compared to the untreated ones in all examined cell lines. CD137-L as an exception thereof however, was significantly downregulated on the WT after irradiation (**Figures 3A–H**).

However, the radioresistant cell lines (WT RR, BR RR) were characterized by a significantly increased expression of especially the immune suppressive ICMs PD-L1, PD-L2 and HVEM in comparison to the respective non-radioresistant clone.

### 3.4 Danger signal Hsp70 is released after irradiation irrespective of the tumor cell clone

**Figure 4:**
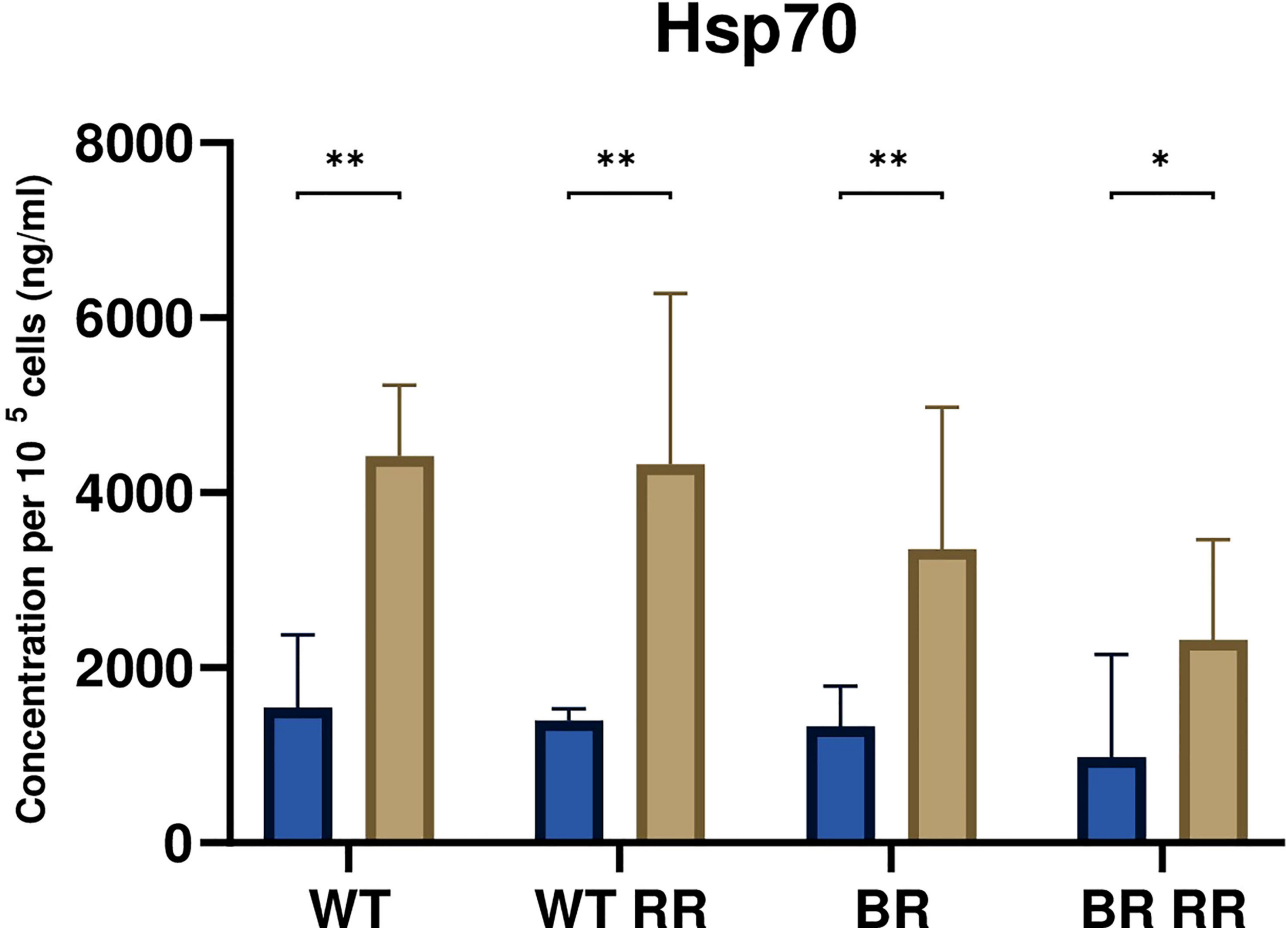
Hsp70 release was significantly increased from irradiated compared to non-irradiated MDA-MB-231 cells. The graph shows the concentration of Hsp70 per 10^5^ cells (ng/ml) in the cell culture supernatant of WT and the WT-derived brain metastasis clone (BR) as well as the radioresistant (RR) clones derived from those cells (WT RR, BR RR), either untreated (blue bars) or after irradiation with 5 × 5.2 Gy (brown bars). Data is presented as median with interquartile range. Data are from six independent experiments. For statistical analysis, a Mann-Whitney U test was performed to compare untreated and treated (5 × 5.2 Gy) cells within one cell line (*p < 0.05, **p < 0.01). Furthermore, a Kruskal-Wallis test with multiple comparisons was used to compare Hsp70 concentrations between the treated WT and its clones.

The release of the damage-associated molecular pattern Hsp70 was significantly increased from the irradiated compared to the respective non-irradiated MDA-MB-231 cells 48 hours after irradiation (**Figure 4**). However, there was no significant difference between the irradiated WT and its different clones.

### 3.5 Neither non-irradiated nor irradiated MDA-MB-231 cells and their supernatants increase the expression of common activation markers on human monocyte-derived DCs

**Figure 5:**
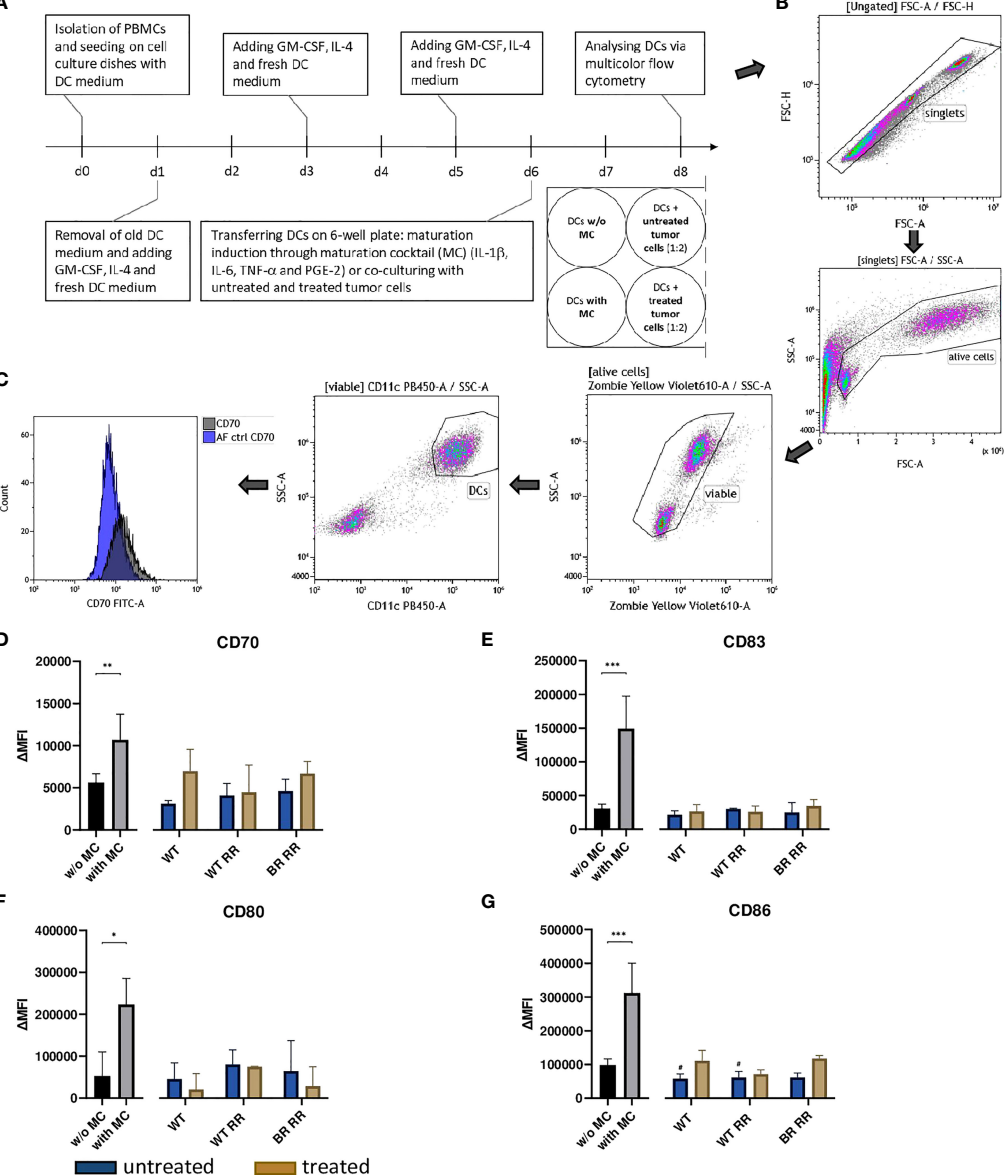
Neither untreated nor treated (5 × 5.2 Gy) MDA-MB-231 cells increased the expression of activation markers on dendritic cells (DCs) 48 hours after co-incubation. **(A)** Human monocyte-derived DCs were differentiated from peripheral blood mononuclear cells (PBMCs) for 5 days before they were co-incubated with untreated and treated wild type (WT) MDA-MB-231 cells or radioresistant (RR) clones. 48 hours later, the expression of common DC activation markers was examined using multicolor flow cytometry. The gating strategy is presented in **(B)** After pre-gating on the singlets, the viable cells were detected. Then, gating on CD11c positive cells identified DCs. CD70 **(D)**, CD83 **(E)**, CD80 **(F)**, CD86 **(G)** expression on the cell surface of DCs is presented in the graphs as ΔMFI. It was calculated by subtracting the Zombie-only-stained samples (AF ctrl) from the respective Zombie-and-antibody-stained samples, here shown exemplarily for CD70 **(C)**. The data is presented as median with interquartile range. Data are from seven independent experiments. For statistical analysis, a Mann-Whitney-U test was used to compare activation marker expression on DCs with and without (w/o) maturation cocktail (MC). Further, a Kruskal-Wallis test was performed to compare DCs w/o MC with DCs which had been co-cultured with either untreated or treated cancer cells, respectively. (*p < 0.05, **p < 0.01, ***p < 0.001, ^#^p < 0.05).

To investigate the potential of treated and untreated RR and non-RR tumor cells to prime DCs, they were co-incubated with human monocyte-derived DCs (**Figures 5A–C**). Incubation of the DCs with the maturation cocktail (MC) led to a significant up-regulation of the four analyzed common activation markers CD70, CD80, CD83 and CD86 (**Figures 5D–G**). However, DCs which were co-incubated with either non-irradiated or irradiated WT, WT RR or BR RR cells and their respective supernatants, did not increase the expression of common activation markers compared to unstimulated, immature DCs (w/o MC).

## 4 Discussion

According to preclinical data, immunogenic cell death induction has been attributed to hypofractionated irradiation schedules (29, 30) which are more and more clinically applied for RT of breast cancer. In contrast to apoptosis which is considered to have a rather suppressive effect on the immune system (31), necrotic cell death is more immune stimulatory because of the release of potentially immunogenic neoantigens and DAMPs such as Hsp70. Necrosis has been reported to be primarily activated by higher doses of ionizing radiation (hypofractionation). It is the desired form of cell death in the context of anti-tumor immune response initiation (18, 32). Unlike other breast cancer subtypes, TNBC is characterized by a rather high TMB. That in turn, leads to the production of neoantigens (33). Consequently, its higher immunogenicity makes it a potential candidate for ICB.

To get first hints about the immunogenic phenotype of MDA-MB-231 breast cancer cells after hypofractionated irradiation and in dependence of their radioresistant properties and tissue origin (e.g. metastatic spread to the brain), cell death forms were determined of the four different clones. Hypofractionated irradiation induced a mixture of apoptosis and necrosis in the four cell lines (**Figure 2**). However, in comparison to the two WT cell lines, both brain-metastasized clones showed to be more sensitive to X-rays leading to strongly apoptosis-dominating cell death. It has been reported that metastatic spread to the brain has an impact on radioresistance of MDA-MB-231 cells as shown by a clonogenic assay (34) which could be due to the influence of the brain microenvironment on gene expression patterns of the tumor cells, as also indicated by the findings of Park et al. (35). Radioresistance was verified as previously described (27). The clonogenic survival (**Figure 1**) confirms that the RR clones have enhanced potential to still form colonies after radiation exposure compared the non-RR clones. Radioresistance was not generally correlated to the capability of tumor cell death induction, as the BR clones had similar amounts of apoptotic and necrotic cells after RT with 5 x 5.2Gy. The relationship between surviving fraction after radiation exposure and the percentage of apoptotic cells at the first days after the same dose of exposure is complex (36). We conclude that radioresistance of the BR cells might also be reflected by cell death forms at later time points than 72 hours and this is already indicated by slightly reduced percentages of necrotic cells of the BR RR clone.

Besides antigenicity, ICD also depends on adjuvanticity (37). Therefore, in the context of cancer cell death, this was exemplarily analyzed by quantifying the Hsp70 concentration in the supernatant of non-irradiated and irradiated tumor cells. In accordance with earlier examinations by Kötter et al., (38). Hsp70 release was significantly increased by irradiated cancer cells in comparison to the respective untreated ones (**Figure 4**). In sufficient quantities, Hsp70 can stimulate the uptake of tumor antigens (39) and further activate dendritic cells (40). Linder et al. suggested that extracellular Hsp70 is released predominantly by active mechanisms and not mainly during cell death (41). Compared to non-irradiated cells, our analyses showed that triple-negative breast cancer cells after radiation therapy have an increased secretion of HSP70 which is independent of the radioresistance. This suggests, as already observed for other tumor entities, that release of HSP70 is mostly connected to necrosis induction (42) of tumor cells (see **Figure 2G**) rather than to radiosensitivity being determined with clonogenic assays. However, radiosensitivity is linked to the immune surface phenotype of the tumor cells, most likely being triggered by activation of DNA sensing pathways in the cytosol of the cells (43).

DCs in general play a key role in T cell priming and therefore provide the basis for T cell-mediated anti-tumor immune responses. To get first hints whether the hypofractionated irradiation of the MDA-MB-231 breast cancer cells affects the priming capabilities of DCs, we examined the expression of DC-specific activation markers after co-incubation with untreated and treated tumor cells. However, although hypofractionated RT induced cell death and the release of Hsp70 in all four cell lines, we did not detect increased expression of any of the investigated activation markers (CD70, CD80, CD83 and CD86) on the surface of the DCs after co-incubation, neither with untreated nor with treated cancer cells in this *in vitro* setting. This suggests that irradiation of breast cancer cells might rather affects the effector phase of anti-tumor immune responses than the priming phase. However more detailed analyses are needed in the future such as how certain DC subsets might be affected. Pilones and colleagues recently discovered that Batf3-dependent conventional dendritic cells type 1 (cDC1) are required for priming RT-induced of tumor-specific CD8+ T cells (44).

Consequently, we analyzed whether the chosen treatment schedule would influence the expression of ICMs on the tumor cells. Most research on immune checkpoint molecules in breast cancer has focused on the PD-1/PD-L1 pathway so far. Monoclonal antibodies (mABs) which antagonize these immune suppressive ICMs and consequently help to restore a potent anti-tumor immune response, have recently led to remarkable long-lasting benefits, but unfortunately just in a minority of (metastatic) cancer patients (24). In this context, higher PD-L1 expression in the tumor has been associated with improved response rates to anti-PD-(L)1 therapies in various cancer types, including TNBC (45–47). Given the predictive value of PD-L1 expression, knowledge about the behaviour of further ICMs in response to irradiation is currently missing but may be beneficial to optimize future radioimmunotherapies (RITs). Therefore, we did not only analyse the expression of PD-L1, but also that of other key ICMs on MDA-MB-231 breast cancer clones.

Consistent with our data, the immune suppressive ICM PD-L1 has been reported to be expressed on the surface of many tumor cells (19, 20, 48, 49). In line with previous *in vitro* and *in vivo* examinations using various cancer cell lines and models, we revealed that it is not just PD-L1 which is upregulated by RT, but rather both immune inhibitory and immune stimulatory (50–53) ICM expression is significantly increased on the surface of TNBC cells following (hypofractionated) irradiation. This has already been demonstrated in other settings and tumor entities (54, 55).

However, a key new finding of our analyses is that particularly immune suppressive checkpoint molecules were significantly more upregulated on the cell surface of radioresistant MDA-MB-231 clones. This indicates for the first time that radioresistant tumour cells do not necessarily have a more stem cell-like phenotype, but might rather suppress the immune system by upregulation of immune suppressive molecules following radiation exposure. This, together with reduction of the dsDNA content in the tumor cells that attenuates the cGAS/STING pathway and consecutively the INFgamma-dependent immune activation (27), contributes to immune suppression. Future analyses will have to deal with connections between RT-induced intracellular modifications and modulations on the tumor cell surface to obtain a complete picture of immune suppressive mechanisms in more radioresistant tumor cell clones. Similarly, Jang et al. found in their single cell RNA-based investigation that PD-L1 expression was increased on radioresistant – based on the radiosensitivity index (RSI) – breast cancer cells. These cells included in particular basal, HER2 and luminal B subtypes and were associated with a higher risk of recurrence (56). Based on our data, we conclude that the immunological phenotype of (breast) cancer cells is strongly shaped by radioresistance. To the best of our knowledge, the underlying mechanisms therefore have not been described in the literature so far and have to be addressed in even more detail in the future, particularly in the context of innovative radiation oncology (57). As TNBC is characterized by a higher infiltration of immune cells it has be suggested that that these patients are more responsive to immunotherapy, but, as already stated, only a minority of these patients benefit from anti-PD-(L)1 monotherapy, which can be improved by adding RT. To achieve further improvement, targeting of additional immune checkpoint molecules should be envisaged (58). Our analyses revealed that HVEM has similar expression patterns such as PD-L1 and PD-L2 and it has been suggested that HVEM negatively correlates with overall survival in breast cancer patients (59). For exploration in clinical application double blockade of the PD-1/PD-L1/2 axis and HVEM in combination with RT should be taken into consideration.

## 5 Conclusion

Basically, there are two main models used to explain tumorigenesis. Both have challenged each other since their existence. On the one hand, there is the cancer stem cell concept which states that so called “cancer stem cells” (CSC) are responsible for cancer development due to their capacity to differentiate into phenotypically diverse cancer cells. Many properties have been attributed to CSCs, amongst others radioresistance. On the other hand, there is the clonal evolution/stochastic model. It assumes that normal cells can acquire distinct mutations over time and become cancer cells (60, 61). Our data indicate that – independent of the CSC concept with its radioresistant stem cells – radioresistant TNBC clones could survive radiotherapy and subsequently, evade the immune response by increased immune suppressive ICM expression. During the last decade it has become evident that the immune system plays an important role in influencing the response to RT treatment and prognosis in many solid tumor entities, including in breast cancer. The most beneficial dose of radiation for induction of anti-tumor immune responses could not be defined until today, but several preclinical, first clinical observation and *in silico* simulations support the hypothesis that hypofractionated RT is the most immunogenic one (62). Following Stereotactic Ablative Body Radiotherapy (SABR), e.g., there is evidence of systemic immune activation in patients with increased PD1 expression (63). Future studies should nevertheless additionally investigate conventional radiation therapy and moderately hypofractionated radiation therapy with regard to radioresistance and immune phenotype of breast cancer cells. In our analyses we aimed to refer to current clinical approaches focusing on more hypofractionated schedules, as already outlined above (10). Generally, the increased immune suppressive ICM expression could then in turn form the basis for recurrence and newly emerging metastases. Therefore, we speculate that the significance of ICB may increase in parallel to the number of experienced radiotherapy sessions and that targeting different ICMs at once might be necessary in breast cancer. We finally want to stress that the key focus was set on the immune phenotype of the radioresistant breast cancer cells in the here presented analyses. Future work will have to focus on even more detailed functional analyses with DC subsets and consecutive T cell activation. Furthermore, analysis of the expression of ICMs in breast cancer specimen tissue microarray with radiation sensitive- and radiation resistant-patient should provide deeper insights how radiosensitivity might be connected to immune phenotypes of breast cancer tumor cells.

## Data availability statement

The raw data supporting the conclusions of this article will be made available by the authors, without undue reservation.

## Ethics statement

The permission to use this LRSC was given by the ethics committee of the Friedrich-Alexander-Universität Erlangen-Nürnberg (ethical approval no. 180_13 B and 48_19 B).

## Author contributions

Conceptualization, UG, MR, BF, KB, and RF; methodology, SG, FM, UG, and BF; validation, MR and UG; formal analysis, SG, FM, AS, and UG; investigation, SG, FG, CR, and JA,; resources, RF, BF, and UG; data curation, CR and FG; writing—original draft preparation, SG and MR; writing—review and editing, MR, UG, FM, and KB; visualization, SG, CR and FG; supervision, MR and UG; project administration, UG and KB; funding acquisition, UG. All authors contributed to the article and approved the submitted version.

## References

[1] Kumar P Aggarwal R. An overview of triple-negative breast cancer. Arch Gynecol Obstet (2016) 293(2):247–69. doi: 10.1007/s00404-015-3859-y 26341644

[2] Nedeljkovic M Damjanovic A. Mechanisms of chemotherapy resistance in triple-negative breast cancer-how we can rise to the challenge. Cells. (2019) 8(9):957. doi: 10.3390/cells8090957 31443516 PMC6770896

[3] Kennecke H Yerushalmi R Woods R Cheang MCU Voduc D Speers CH. Metastatic behavior of breast cancer subtypes. J Clin Oncol (2010) 28(20):3271–7. doi: 10.1200/JCO.2009.25.9820 20498394

[4] Early Breast Cancer Trialists' Collaborative G Darby S McGale P Correa C Taylor C Arriagada R. Effect of radiotherapy after breast-conserving surgery on 10-year recurrence and 15-year breast cancer death: meta-analysis of individual patient data for 10,801 women in 17 randomised trials. Lancet (2011) 378(9804):1707–16. doi: 10.1016/S0140-6736(11)61629-2 PMC3254252 22019144

[5] Ebctcg McGale P Taylor C Correa C Cutter D Duane F. Effect of radiotherapy after mastectomy and axillary surgery on 10-year recurrence and 20-year breast cancer mortality: meta-analysis of individual patient data for 8135 women in 22 randomised trials. Lancet (2014) 383(9935):2127–35. 10.1016/S0140-6736(14)60488-8 PMC5015598 24656685

[6] Krug D Baumann R Combs SE Duma MN Dunst J Feyer P. Moderate hypofractionation remains the standard of care for whole-breast radiotherapy in breast cancer: Considerations regarding FAST and FAST-forward. Strahlenther Onkol (2021) 197(4):269–80. doi: 10.1007/s00066-020-01744-3 PMC7841378 33507331

[7] Whelan TJ Pignol JP Levine MN Julian JA MacKenzie R Parpia S. Long-term results of hypofractionated radiation therapy for breast cancer. N Engl J Med (2010) 362(6):513–20. doi: 10.1056/NEJMoa0906260 20147717

[8] Group ST Bentzen SM Agrawal RK Aird EG Barrett JM Barrett-Lee PJ. The UK standardisation of breast radiotherapy (START) trial b of radiotherapy hypofractionation for treatment of early breast cancer: a randomised trial. Lancet. (2008) 371(9618):1098–107. 10.1016/S0140-6736(08)60348-7 PMC2277488 18355913

[9] Shaitelman SF Schlembach PJ Arzu I Ballo M Bloom ES Buchholz D. Acute and short-term toxic effects of conventionally fractionated vs hypofractionated whole-breast irradiation: A randomized clinical trial. JAMA Oncol (2015) 1(7):931–41. doi: 10.1001/jamaoncol.2015.2666 PMC4635441 26247543

[10] Murray Brunt A Haviland JS Wheatley DA Sydenham MA Alhasso A Bloomfield DJ. Hypofractionated breast radiotherapy for 1 week versus 3 weeks (FAST-forward): 5-year efficacy and late normal tissue effects results from a multicentre, non-inferiority, randomised, phase 3 trial. Lancet (2020) 395(10237):1613–26. doi: 10.1016/S0140-6736(20)30932-6 PMC7262592 32580883

[11] Mondini M Levy A Meziani L Milliat F Deutsch E. Radiotherapy-immunotherapy combinations - perspectives and challenges. Mol Oncol (2020) 14(7):1529–37. doi: 10.1002/1878-0261.12658 PMC7332212 32112478

[12] Saleiro D Platanias LC. Interferon signaling in cancer. non-canonical pathways and control of intracellular immune checkpoints. Semin Immunol (2019) 43:101299. doi: 10.1016/j.smim.2019.101299 31771762 PMC8177745

[13] Patel DJ Yu Y Xie W. cGAMP-activated cGAS-STING signaling: its bacterial origins and evolutionary adaptation by metazoans. Nat Struct Mol Biol (2023) 30, 245–260. doi: 10.1038/s41594-023-00933-9 PMC11749898 36894694

[14] Pellegrino B Musolino A Llop-Guevara A Serra V De Silva P Hlavata Z. Homologous recombination repair deficiency and the immune response in breast cancer: A literature review. Transl Oncol (2020) 13(2):410–22. doi: 10.1016/j.tranon.2019.10.010 PMC6948367 31901781

[15] Vasiyani H Mane M Rana K Shinde A Roy M Singh J. DNA Damage induces STING mediated IL-6-STAT3 survival pathway in triple-negative breast cancer cells and decreased survival of breast cancer patients. Apoptosis (2022) 27(11-12):961–78. doi: 10.1007/s10495-022-01763-8 36018392

[16] Ruckert M Flohr AS Hecht M Gaipl US. Radiotherapy and the immune system: More than just immune suppression. Stem Cells (2021) 39(9):1155–65. doi: 10.1002/stem.3391 33961721

[17] Galluzzi L Vitale I Warren S Adjemian S Agostinis P Martinez AB. Consensus guidelines for the definition, detection and interpretation of immunogenic cell death. J Immunother Cancer (2020) 8(1). doi: 10.1136/jitc-2019-000337corr1 PMC7064135 32209603

[18] Frey B Ruckert M Deloch L Ruhle PF Derer A Fietkau R. Immunomodulation by ionizing radiation-impact for design of radio-immunotherapies and for treatment of inflammatory diseases. Immunol Rev (2017) 280(1):231–48. doi: 10.1111/imr.12572 29027224

[19] Iwai Y Ishida M Tanaka Y Okazaki T Honjo T Minato N. Involvement of PD-L1 on tumor cells in the escape from host immune system and tumor immunotherapy by PD-L1 blockade. Proc Natl Acad Sci USA (2002) 99(19):12293–7. doi: 10.1073/pnas.192461099 PMC129438 12218188

[20] Wintterle S Schreiner B Mitsdoerffer M Schneider D Chen L Meyermann R. Expression of the B7-related molecule B7-H1 by glioma cells: a potential mechanism of immune paralysis. Cancer Res (2003) 63(21):7462–7. 14612546

[21] Esteva FJ Hubbard-Lucey VM Tang J Pusztai L. Immunotherapy and targeted therapy combinations in metastatic breast cancer. Lancet Oncol (2019) 20(3):e175–e86. doi: 10.1016/S1470-2045(19)30026-9 30842061

[22] Thomas R Al-Khadairi G Decock J. Immune checkpoint inhibitors in triple negative breast cancer treatment: Promising future prospects. Front Oncol (2020) 10:600573. doi: 10.3389/fonc.2020.600573 33718107 PMC7947906

[23] Adams S Schmid P Rugo HS Winer EP Loirat D Awada A. Pembrolizumab monotherapy for previously treated metastatic triple-negative breast cancer: cohort a of the phase II KEYNOTE-086 study. Ann Oncol (2019) 30(3):397–404. doi: 10.1093/annonc/mdy517 30475950

[24] Ho AY Barker CA Arnold BB Powell SN Hu ZI Gucalp A. A phase 2 clinical trialassessing theefficacy and safety of pembrolizumab and radiotherapy in patients with metastatic triple-negative breast cancer. Cancer (2020) 126(4):850–60. doi: 10.1002/cncr.32599 31747077

[25] Franken NA Rodermond HM Stap J Haveman J van Bree C. Clonogenic assay of cells in vitro. Nat Protoc (2006) 1(5):2315–9. doi: 10.1038/nprot.2006.339 17406473

[26] Brix N Samaga D Belka C Zitzelsberger H Lauber K. Analysis of clonogenic growth in vitro. Nat Protoc (2021) 16(11):4963–91. doi: 10.1038/s41596-021-00615-0 34697469

[27] Meyer F Engel AM Krause AK Wagner T Poole L Dubrovska A. Efficient DNA repair mitigates replication stress resulting in less immunogenic cytosolic DNA in radioresistant breast cancer stem cells. Front Immunol (2022) 13:765284. doi: 10.3389/fimmu.2022.765284 35280989 PMC8913591

[28] Yoneda T Williams PJ Hiraga T Niewolna M Nishimura R. A bone-seeking clone exhibits different biological properties from the MDA-MB-231 parental human breast cancer cells and a brain-seeking clone *in vivo* and *in vitro* . J Bone Miner Res (2001) 16(8):1486–95. doi: 10.1359/jbmr.2001.16.8.1486 11499871

[29] Vanpouille-Box C Alard A Aryankalayil MJ Sarfraz Y Diamond JM Schneider RJ. DNA Exonuclease Trex1 regulates radiotherapy-induced tumour immunogenicity. Nat Commun (2017) 8:15618. doi: 10.1038/ncomms15618 28598415 PMC5472757

[30] Deng L Liang H Xu M Yang X Burnette B Arina A. STING-dependent cytosolic DNA sensing promotes radiation-induced type I interferon-dependent antitumor immunity in immunogenic tumors. Immunity. (2014) 41(5):843–52. doi: 10.1016/j.immuni.2014.10.019 PMC5155593 25517616

[31] Voll RE Herrmann M Roth EA Stach C Kalden JR Girkontaite I. Immunosuppressive effects of apoptotic cells. Nature (1997) 390(6658):350–1. doi: 10.1038/37022 9389474

[32] McKelvey KJ Hudson AL Back M Eade T Diakos CI. Radiation, inflammation and the immune response in cancer. Mamm Genome (2018) 29(11-12):843–65. doi: 10.1007/s00335-018-9777-0 PMC6267675 30178305

[33] Narang P Chen M Sharma AA Anderson KS Wilson MA. The neoepitope landscape of breast cancer: implications for immunotherapy. BMC Cancer (2019) 19(1):200. doi: 10.1186/s12885-019-5402-1 30832597 PMC6399957

[34] Smart D Garcia-Glaessner A Palmieri D Wong-Goodrich SJ Kramp T Gril B. Analysis of radiation therapy in a model of triple-negative breast cancer brain metastasis. Clin Exp Metastasis (2015) 32(7):717–27. doi: 10.1007/s10585-015-9739-9 PMC7743910 26319493

[35] Park ES Kim SJ Kim SW Yoon SL Leem SH Kim SB. Cross-species hybridization of microarrays for studying tumor transcriptome of brain metastasis. Proc Natl Acad Sci U S A (2011) 108(42):17456–61. doi: 10.1073/pnas.1114210108 PMC3198333 21987811

[36] Dunne AL Price ME Mothersill C McKeown SR Robson T Hirst DG. Relationship between clonogenic radiosensitivity, radiation-induced apoptosis and DNA damage/repair in human colon cancer cells. Br J Cancer (2003) 89(12):2277–83. doi: 10.1038/sj.bjc.6601427 PMC2395286 14676806

[37] Galluzzi L Vitale I Aaronson SA Abrams JM Adam D Agostinis P. Molecular mechanisms of cell death: recommendations of the nomenclature committee on cell death 2018. Cell Death Differ (2018) 25(3):486–541. doi: 10.1038/s41418-017-0012-4 29362479 PMC5864239

[38] Kötter B Frey B Winderl M Rubner Y Scheithauer H Sieber R. The in vitro immunogenic potential of caspase-3 proficient breast cancer cells with basal low immunogenicity is increased by hypofractionated irradiation. Radiat Oncol (2015) 10(1). 10.1186/s13014-015-0506-5 PMC4573696 26383236

[39] Galluzzi L Buqué A Kepp O Zitvogel L Kroemer G. Immunogenic cell death in cancer and infectious disease. Nat Rev Immunol (2016) 17(2):97–111. 27748397 10.1038/nri.2016.107

[40] Schildkopf P Frey B Ott OJ Rubner Y Multhoff G Sauer R. Radiation combined with hyperthermia induces HSP70-dependent maturation of dendritic cells and release of pro-inflammatory cytokines by dendritic cells and macrophages. Radiother Oncol (2011) 101(1):109–15. doi: 10.1016/j.radonc.2011.05.056 21704416

[41] Linder M Pogge von Strandmann E. The role of extracellular HSP70 in the function of tumor-associated immune cells. Cancers (Basel) (2021) 13(18):4721. doi: 10.3390/cancers13184721 34572948 PMC8466959

[42] Rubner Y Muth C Strnad A Derer A Sieber R Buslei R. Fractionated radiotherapy is the main stimulus for the induction of cell death and of Hsp70 release of p53 mutated glioblastoma cell lines. Radiat Oncol (2014) 9(1):89. 24678590 10.1186/1748-717X-9-89 PMC3994240

[43] Ye Z Shi Y Lees-Miller SP Tainer JA. Function and molecular mechanism of the DNA damage response in immunity and cancer immunotherapy. Front Immunol (2021) 12:797880. doi: 10.3389/fimmu.2021.797880 34970273 PMC8712645

[44] Pilones KA Charpentier M Garcia-Martinez E Daviaud C Kraynak J Aryankalayil J. Radiotherapy cooperates with IL15 to induce antitumor immune responses. Cancer Immunol Res (2020) 8(8):1054–63. doi: 10.1158/2326-6066.CIR-19-0338 PMC7415682 32532811

[45] Cortes J Cescon DW Rugo HS Nowecki Z Im SA Yusof MM. Pembrolizumab plus chemotherapy versus placebo plus chemotherapy for previously untreated locally recurrent inoperable or metastatic triple-negative breast cancer (KEYNOTE-355): a randomised, placebo-controlled, double-blind, phase 3 clinical trial. Lancet. (2020) 396(10265):1817–28. doi: 10.1016/S0140-6736(20)32531-9 33278935

[46] Dirix LY Takacs I Jerusalem G Nikolinakos P Arkenau HT Forero-Torres A. Avelumab, an anti-PD-L1 antibody, in patients with locally advanced or metastatic breast cancer: a phase 1b JAVELIN solid tumor study. Breast Cancer Res Treat (2018) 167(3):671–86. doi: 10.1007/s10549-017-4537-5 PMC5807460 29063313

[47] Emens LA Cruz C Eder JP Braiteh F Chung C Tolaney SM. Long-term clinical outcomes and biomarker analyses of atezolizumab therapy for patients with metastatic triple-negative breast cancer: A phase 1 study. JAMA Oncol (2019) 5(1):74–82. doi: 10.1001/jamaoncol.2018.4224 30242306 PMC6439773

[48] Mittal D Gubin MM Schreiber RD Smyth MJ. New insights into cancer immunoediting and its three component phases–elimination, equilibrium and escape. Curr Opin Immunol (2014) 27:16–25. doi: 10.1016/j.coi.2014.01.004 24531241 PMC4388310

[49] Dong H Strome SE Salomao DR Tamura H Hirano F Flies DB. Tumor-associated B7-H1 promotes T-cell apoptosis: a potential mechanism of immune evasion. Nat Med (2002) 8(8):793–800. doi: 10.1038/nm730 12091876

[50] Dovedi SJ Adlard AL Lipowska-Bhalla G McKenna C Jones S Cheadle EJ. Acquired resistance to fractionated radiotherapy can be overcome by concurrent PD-L1 blockade. Cancer Res (2014) 74(19):5458–68. 10.1158/0008-5472.CAN-14-1258 25274032

[51] Sato H Niimi A Yasuhara T Permata TBM Hagiwara Y Isono M. DNA Double-strand break repair pathway regulates PD-L1 expression in cancer cells. Nat Commun (2017) 8(1):1751. 29170499 10.1038/s41467-017-01883-9 PMC5701012

[52] Wu C-T Chen W-C Chang Y-H Lin W-Y Chen M-F. The role of PD-L1 in the radiation response and clinical outcome for bladder cancer. Sci Rep (2016) 6(1). 10.1038/srep19740 PMC4726250 26804478

[53] Derer A Spiljar M Baumler M Hecht M Fietkau R Frey B. Chemoradiation increases PD-L1 expression in certain melanoma and glioblastoma cells. Front Immunol (2016) 7:610. 28066420 10.3389/fimmu.2016.00610 PMC5177615

[54] Hader M Savcigil DP Rosin A Ponfick P Gekle S Wadepohl M. Differences of the immune phenotype of breast cancer cells after ex vivo hyperthermia by warm-water or microwave radiation in a closed-loop system alone or in combination with radiotherapy. Cancers (2020) 12(5):1082. 32349284 10.3390/cancers12051082 PMC7281749

[55] Wimmer S Deloch L Hader M Derer A Grottker F Weissmann T. Hypofractionated radiotherapy upregulates several immune checkpoint molecules in head and neck squamous cell carcinoma cells independently of the HPV status while ICOS-l is upregulated only on HPV-positive cells. Int J Mol Sci (2021) 22(17):9114. 34502022 10.3390/ijms22179114 PMC8430967

[56] Jang BS Han W Kim IA. Tumor mutation burden, immune checkpoint crosstalk and radiosensitivity in single-cell RNA sequencing data of breast cancer. Radiother Oncol (2020) 142:202–9. doi: 10.1016/j.radonc.2019.11.003 31767471

[57] Krug D Hecht M Ebert N Mäurer M Fleischmann DF Fokas E. Innovative radiation oncology together - precise, personalized, human : Vision 2030 for radiotherapy & radiation oncology in Germany. Strahlenther Onkol (2021) 197(12):1043–8. doi: 10.1007/s00066-021-01843-9 PMC8604860 34515820

[58] Yi H Li Y Tan Y Fu S Tang F Deng X. Immune checkpoint inhibition for triple-negative breast cancer: Current landscape and future perspectives. Front Oncol (2021) 11:648139. 34094935 10.3389/fonc.2021.648139 PMC8170306

[59] Tsang JYS Chan KW Ni YB Hlaing T Hu J Chan SK. Expression and clinical significance of herpes virus entry mediator (HVEM) in breast cancer. Ann Surg Oncol (2017) 24(13):4042–50. 10.1245/s10434-017-5924-1 28612127

[60] Nie D Bartram I Jeschke JM. Do cancer stem cells exist? a pilot study combining a systematic review with the hierarchy-of-hypotheses approach. PloS One (2019) 14(12). 10.1371/journal.pone.0225898 PMC6910685 31834886

[61] Arnold CR Mangesius J Skvortsova I-I Ganswindt U. The role of cancer stem cells in radiation resistance. Front Oncol (2020) 10. 10.3389/fonc.2020.00164 PMC7044409 32154167

[62] Poleszczuk J Enderling H. The optimal radiation dose to induce robust systemic anti-tumor immunity. Int J Mol Sci (2018) 19(11). 10.3390/ijms19113377 PMC6275030 30380596

[63] David S Tan J Siva S Karroum L Savas P Loi S. Combining radiotherapy and immunotherapy in metastatic breast cancer: Current status and future directions. Biomedicines (2022) 10(4):821. doi: 10.3390/biomedicines10040821 35453571 PMC9024725

